# Collaboration between HPMC and NaCMC in order to Reach the Polymer Critical Point in Theophylline Hydrophilic Matrices

**DOI:** 10.1100/2012/171292

**Published:** 2012-08-01

**Authors:** L. Contreras, L. M. Melgoza, A. Aguilar-de-Leyva, I. Caraballo

**Affiliations:** ^1^Department of Pharmacy and Pharmaceutical Technology, University of Seville, C/Profesor García González, 2, 41012 Seville, Spain; ^2^Departamento de Sistemas Biológicos, Universidad Autónoma Metropolitana Xochimilco, Calzada del Hueso 1100, Col. Villa Quietud, Delegación Coyoacán, 04960 México, DF, Mexico

## Abstract

Percolation theory has been applied in order to study the existence of critical points as well as the possibility to find a “combined percolation threshold” for ternary hydrophilic matrices prepared with HPMC, NaCMC, and theophylline. For this purpose, different batches of ternary as well as binary hydrophilic matrices have been prepared. Critical points have been found for binary hydrophilic matrices between 21.5 and 31.3% (v/v) of HPMC and between 39 and 54% (v/v) of NaCMC, respectively. In a previous work carried out with the same polymers but a much more soluble drug (KCl), it was demonstrated the existence of a partial collaboration between the polymers in order to establish the gel layer. In this work, it has been observed for the first time the need of a minimum concentration of one of the matrix-forming polymer (between 10 and 20% v/v, approximately) for establishing an effective collaboration.

## 1. Introduction

Hydrophilic matrices consist of a mixture of one or more active ingredients with one or more gel-forming agents. This mixture is usually compressed into tablets [1]. In order to formulate a successful hydrophilic matrix tablet, a polymer substance that will wet, hydrate, and swell forming a gelatinous layer fast enough to avoid the disintegration of the tablet and to protect the tablet content from dissolving during the initial wetting and hydration phases must be selected [2]. For this purpose, different cellulosic derivatives like hydroxypropylmethylcellulose (HPMC), hydroxypropylcellulose (HPC), sodium carboxymethylcellulose (NaCMC), and methylcellulose (MC) or their combinations have been extensively used in the preparation of matrix tablets [1–4]. 

Drug release from these systems is controlled by a combination of several physical processes which include, but are not limited to diffusion, polymer swelling, erosion, and dissolution [5]. The gelling agent is the element of the formulation that is most responsible for the formation, by hydration, of a diffusion- and erosion-resistant gel layer [6]. This gel layer is a diffusional barrier that controls the water uptake and the release of the dissolved drug. Water soluble drugs are released primarily by diffusion of dissolved drug molecules across the gel layer, while poor water soluble drugs are released predominantly by an erosion mechanism. The contribution of each mechanism to the overall drug release process is influenced both by the drug solubility and also by the physical and mechanical properties of the gel barrier formed around the tablet [7]. 

Percolation theory studies disordered systems where the components are randomly distributed in a lattice. This theory deals with the number and properties of clusters of occupied sites in real or virtual lattices. A cluster is defined as a group of neighboring-occupied sites in the lattice. [8]. When clusters are isolated, they are called finite. On the other hand, when a cluster percolates a lattice, that is, when a cluster spans the length, breadth, and height of a lattice it is considered an infinite cluster, in an infinite theoretical lattice or a percolating cluster, in a real system. The concentration of a component at which there is the maximum probability that this component starts to percolate a system is named the percolation threshold, *p*_*c*_, of this component. In a binary tablet, two percolation thresholds can be found; the drug and the excipient percolation thresholds_._

Through the application of percolation theory, it is possible to gain new insights into well known problems of pharmaceutical technology, such as, dosage form design, characterization of dosage forms, unit operations in production and drug release properties of matrix systems [9]. This theory has proved to be useful, especially for the characterization and design of binary dosage forms in which the changes in the dissolution kinetics and water uptake can be explained using percolation [10–13]. However, drug delivery systems usually contain more than two components. Different works have studied the possibility to apply the percolation theory, simplifying a multicomponent system to a binary one (on the basis of a common property, as for example solubility or hydrophilicity), finding the existence of a combined percolation threshold [14, 15]. 

Caraballo et al. defined in 1996 a combined percolation threshold of two components in ternary systems, as the volume fraction at which these components, jointly considered, start to percolate the sample. 

In a previous work, the dissolution behaviour and water uptake of ternary hydrophilic matrices containing hydroxypropylmethylcellulose (HPMC), sodium carboxymethylcellulose (NaCMC) and an extremely water soluble substance as potassium chloride (KCl) have been analyzed for the first time applying the concepts of the percolation theory. In this work, the existence of an excipient critical barrier (in matrices prepared with each single polymer as well as matrices containing mixtures of the two polymers) was observed, and it was demonstrated that the excipients showed a partial collaboration between them in order to establish the gel layer [16]. 

Taking into account that the solubility of the drug can strongly influence the processes leading to obtaining a consistent gel layer, the present study investigates the collaboration pattern between HPMC and NaCMC using a model drug with a solubility much more moderated than KCl. For this purpose the drug release and water uptake behaviour of ternary hydrophilic matrix systems manufactured using HPMC, NaCMC, and theophylline as model drug have been studied. Percolation theory is applied in order to study the existence of critical points as well as the possibility to find a “combined percolation threshold” for the polymers. The results will be compared with those obtained for ternary systems containing KCl as well as multicomponent matrix systems previously studied.

## 2. Materials and Methods

### 2.1. Materials

In order to manufacture the hydrophilic matrix tablets, theophylline anhydrous (mean particle size 217.24 ± 82.64 *μ*m) (Roig Farma, Tarrasa Barcelona, Spain) and the 150–200 *μ*m granulometric fraction of the matrix-forming polymers hydroxypropylmethylcellulose (HPMC K4M, Colorcon, S.A., Spain) and sodium carboxymethylcellulose (NaCMC Akucell AF 3295 Safic-Alcan, Spain) have been employed.

### 2.2. Preparation of the Hydrophilic Matrix Tablets. 

The true density was determined using an air pycnometer (Quantachrome mod. Stereopycnometer spy-3) as 1.802, 1.285, and 1.376 g/cm^3^ for theophylline, HPMC K4M, and NaCMC, respectively.

**Figure 1:**
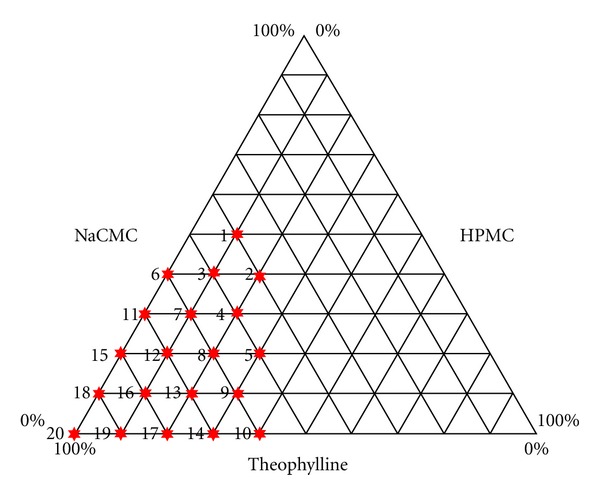
Composition in % w/w of the studied batches containing theophylline and different amounts of NaCMC and HPMC.

The components were mixed using a Turbula mixer (Basel, Switzerland). The mixing time (3 min) was validated. Then, binary and ternary hydrophilic matrix systems were prepared varying the percentages of the components with a drug dose of 250 mg (see Figure 1). For example, batch 1 contains 40% w/w theophylline, 10% w/w HPMC, and 50% NaCMC.

Cylindrical tablets with a diameter of 12 mm were obtained using an eccentric machine Bonals A-300 (Barcelona, Spain) by direct compression, applying the maximum compression force accepted by the formulation.

### 2.3. Dissolution Studies

Dissolution studies were carried out in 900 mL of deionizated water, using the apparatus II proposed by USP 26 (Sotax AT7) at 37°C + 0.5°C and 100 rpm. This test was performed in triplicate. The drug released was detected by UV spectroscopy at 272 nm.

In order to find a possible change on the drug release kinetics, the fit of the data corresponding to 5–70% drug release to the zero order (1), Higuchi [17], (2) Korsmeyer [18], (3) and Peppas and Sahlin [19]. Equation (4) models were studied.

(1) $$\begin{array}{c} \frac{Q_{t}}{Q_{\infty }}=kt, \end{array}$$

(2) $$\begin{array}{c} \frac{Q_{t}}{Q_{\infty }}=bt^{1/2}, \end{array}$$

(3) $$\begin{array}{c} \frac{Q_{t}}{Q_{\infty }}=kt^{n}, \end{array}$$

(4) $$\begin{array}{c} \frac{Q_{t}}{Q_{\infty }}=k_{d}t^{m}+k_{r}t^{2m}, \end{array}$$

where: *Q*_*t*_/*Q*_*∞*_ = fraction of drug released, *b* = Higuchi's slope, *k* = kinetic constant of the Korsmeyer model, *n* = diffusional exponent which depends on the release mechanism and the shape of the device, *k*_*d*_ = diffusional constant of Peppas and Sahlin model, *k*_*r*_ = relaxation constant of Peppas and Sahlin model, *m* = diffusional exponent which depends on the geometrical shape of the releasing device through its aspect ratio.

### 2.4. Water Uptake Studies

The water uptake studies were performed using the modified Enslin apparatus. The water uptake through one side of the tablet at each time was read from a precision balance (Scaltec SBC 31) linked to a chart recorder and a personal computer, during 12 hours, employing deionizated water. This test was performed in three replicates. 

The Davidson and Peppas model [20] (5) was applied to these data to study the rate of water uptake:

(5) $$\begin{array}{c} w=k_{s}t^{n}, \end{array}$$

where: *w* = weight gain of the swelled matrix (water/dry polymer), *k*_*s*_= kinetic constant of water penetration, *t* = penetration time, *n* = exponent which depends on the water penetration mechanism. 

### 2.5. Estimation of the Percolation Threshold

In order to estimate the percolation threshold, the behaviour of several properties of the system has been studied. 

The dissolution results were employed to estimate the excipient percolation threshold of the HPMC and NaCMC formulations. An abrupt change in the release profiles or in the kinetic parameters indicates a change in the release behavior and could be indicative of a phase transition related to the presence of a percolation threshold of one component of the formulation. 

The results of the water uptake studies have also been employed as an auxiliary technique to estimate the percolation threshold of the obtained matrices.

 Furthermore, the behaviour of the kinetic parameter *Higuchi's slope* “*b*” was studied as a function of the volumetric fraction of the components of the formulation at time zero.

The percentage of the drug released and the amount of the drug released normalized with the volumetric fraction of the drug (Q/F_Theophylline_) has been plotted versus the square root of the time to show the different behavior of the matrices below and above the polymer percolation threshold.

In order to study the existence of combined percolation thresholds on the ternary hydrophilic matrix systems, the obtained data were plotted using the Matlab program.

## 3. Results and Discussion

As it can be observed in Figure 1, binary and ternary formulations were compressed employing the model drug theophylline. The dissolution and water uptake behaviour of the binary matrix systems theophylline and HPMC and Theophylline-NaCMC and the corresponding critical points were studied in a first step. After that, the ternary systems theophylline, HPMC, and NaCMC were studied. 

### 3.1. In Vitro Drug Release Studies for Binary Hydrophilic Matrix Systems Theophylline and HPMC

**Figure 2:**
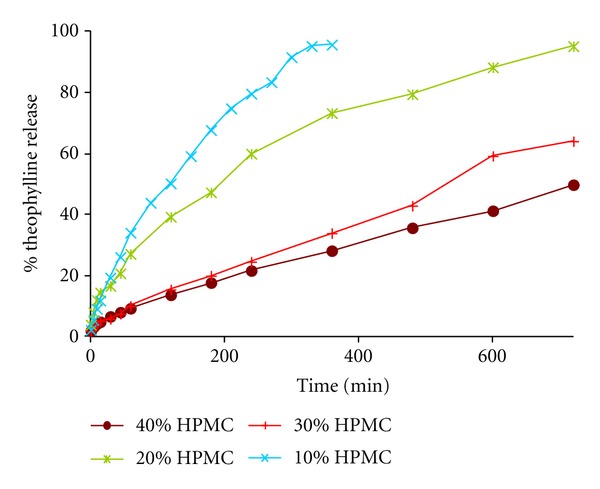
Dissolution profiles of the binary hydrophilic matrix systems elaborated with theophylline and HPMC.

The dissolution studies for the binary theophylline and HPMC systems are shown in Figure 2. Firstly, the drug release profiles were analyzed visually, observing an important change between 20 and 30% (w/w) HPMC. Secondly, the kinetic parameters were analyzed, confirming the change mentioned above.  Table 1 shows the values obtained from zero order, Higuchi's, Korsmeyer, and Peppas and Sahlin models.

As it can be observed, the Higuchi's slope, *b*, shows and important increase between 20 and 30% (w/w) HPMC (from 2.406 to 4.693% min^−1/2^ for batches 14 and 17) as well as the Korsmeyer's constant rate, *k*, (from 0.354 to 1.696% min^−*n*^ for batches 14 and 17) for these binary systems. 

The values of the Peppas and Sahlin constants confirm this change on the drug release mechanism. The diffusion constant, *k*_*d*_, increases from 0.524 to 2.311% min^−*m*^ and the relaxational constant, *k*_*r*_, changes from 0.159 to 0.254% min^−2*m*^. Therefore, both drug release profiles and the kinetic analysis confirm the existence of a critical point for the binary HPMC/theophylline matrices between 20 and 30% (w/w) HPMC, corresponding to 21.5–31.4% (v/v) HPMC.

### 3.2. In Vitro Drug Release Studies for Binary Hydrophilic Matrix Systems Theophylline and CMCNa

**Figure 3:**
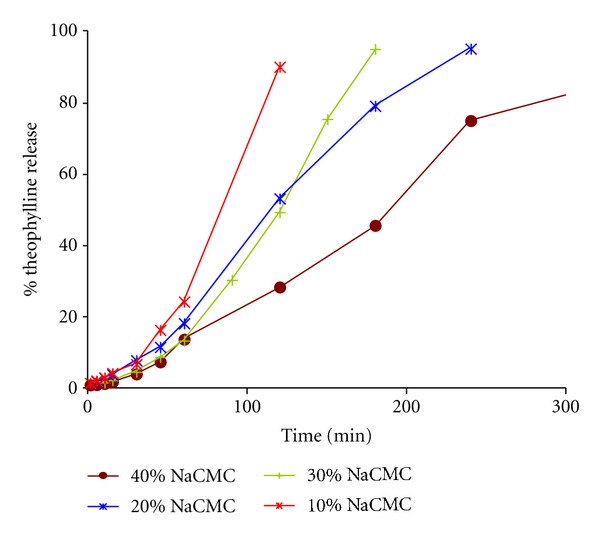
Dissolution profiles of the binary hydrophilic matrix systems elaborated with theophylline and NaCMC.

The results of the dissolution studies for the binary systems prepared with theophylline and NaCMC are shown in Figure 3, where it can observe a change in the drug release profiles between 30 and 40% (w/w) NaCMC. The kinetic analysis (Table 1) of the drug release profiles from the matrix systems studied was performed employing the models mentioned in the previous section. The data of the Peppas and Sahlin model show that in all the batches the drug release process is mainly governed by the relaxation of the polymer chains or by the erosion of the matrix system, since the relaxation constant, *k*_*r*_, is much higher than the diffusional constant, *k*_*d*_. This is in agreement with the general behavior of NaCMC, being more erodible than HPMC as matrix forming polymer.

Looking at the kinetic data, the Higuchi's slope, *b*, shows an important decrease (from 7.934 to 6.804% min^−1/2^) and the Korsmeyer's constant rate, *k*, an increase (from 0.002 to 0.058% min^−*n*^) between the batches containing 30 and 40% w/w of NaCMC. Furthermore, the Korsmeyer's exponent and the diffusional constant of Peppas and Sahlin almost duplicate their values between these lots. This analysis confirms the existence of a critical point between 30–40% (w/w) NaCMC, corresponding to the 39–54% (v/v) of NaCMC.

On the other hand, the data obtained from the profiles containing between 70 and 80% (w/w) of theophylline shows that the drug release mechanism can be described by the zero order model. The batch with a higher concentration of drug (90% (w/w)) shows that the drug release is mainly due to the erosion of the system.

### 3.3. In Vitro Drug Release Studies for Ternary Hydrophilic Matrix Systems Theophylline, HPMC, and NaCMC

Ternary hydrophilic matrix systems were prepared in order to study the drug release behavior of these systems, investigating the existence of a critical excipient barrier and the possible differences with this found in a previous work using KCl as model drug [16]. 

**Figure 4:**
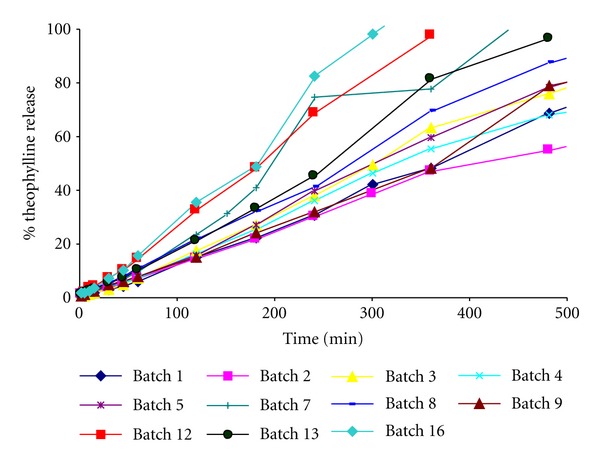
Dissolution profiles of the ternary hydrophilic matrix systems elaborated with theophylline, HPMC, and NaCMC.

Figure 4 shows the drug release profiles obtained for the studied matrices. Two different behaviors can be observed. On one hand, faster drug release profiles can be appreciated for batches 7, 12, and 16. Peppas and Sahlin model showed higher values for the relaxation constant with respect to the diffusional constant, meaning that the principal mechanism for the drug release is the relaxation or erosion of the polymer chains. 

On the other hand, batches 1, 2, 3, 4, 5, 8, and 9 show slower drug release profiles and a better fit of the data to the zero order release kinetics, being batch 13 intermediate between these two behaviours.

Considering in general the obtained ternary matrices, it can be appreciated values of n near to 1 for the fit of the data of these batches to the Korsmeyer model. For the Peppas and Sahlin model, the relaxation constant shows higher values than the diffusional constant (see Table 1).

### 3.4. Water Uptake Studies

Water uptake studies were carried out in the modified Enslin apparatus. For the theophylline-HPMC binary mixtures (batches 10, 14, 17, and 19), according to the increase in the swelling constant *k*_*s*_, a transition can be located between 10% and 20% (w/w) HPMC (10.76–21.55% v/v). The binary systems theophylline and NaCMC showed a change from a fast swelling constant *k*_*s*_ (40% (w/w) NaCMC) to a slow swelling constant *k*_*s*_ (30% (w/w) NaCMC). According to these water uptake profiles, the critical range for this property can be located between 30–40% (w/w) NaCMC (see Table 2).

In the case of NaCMC, the percolation threshold for the water uptake agrees with the percolation threshold obtained for the drug release. However, in the case of HPMC, the water uptake percolation threshold is different from the percolation threshold obtained for the drug release. This fact can be explained taking into account that the water uptake is measured only through one side of the tablet, while the drug release takes place through all the faces of the tablet.

**Figure 5:**
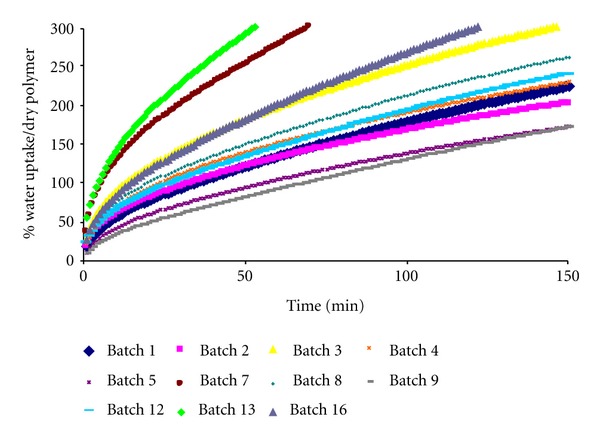
Water uptake profiles of the ternary hydrophilic matrix systems containing Theophylline, HPMC, and NaCMC.

 It can be appreciated that if the concentration of HPMC on the matrix system increases, the water uptake rate decreases on the batches 1, 2, 3, 4, 5, 7, 8, and 9 (see Figure 5).

### 3.5. Estimation of Excipient Percolation Thresholds

It has been demonstrated that the excipient percolation threshold influences the drug release from hydrophilic matrix systems [16, 21]. 

**Figure 6:**
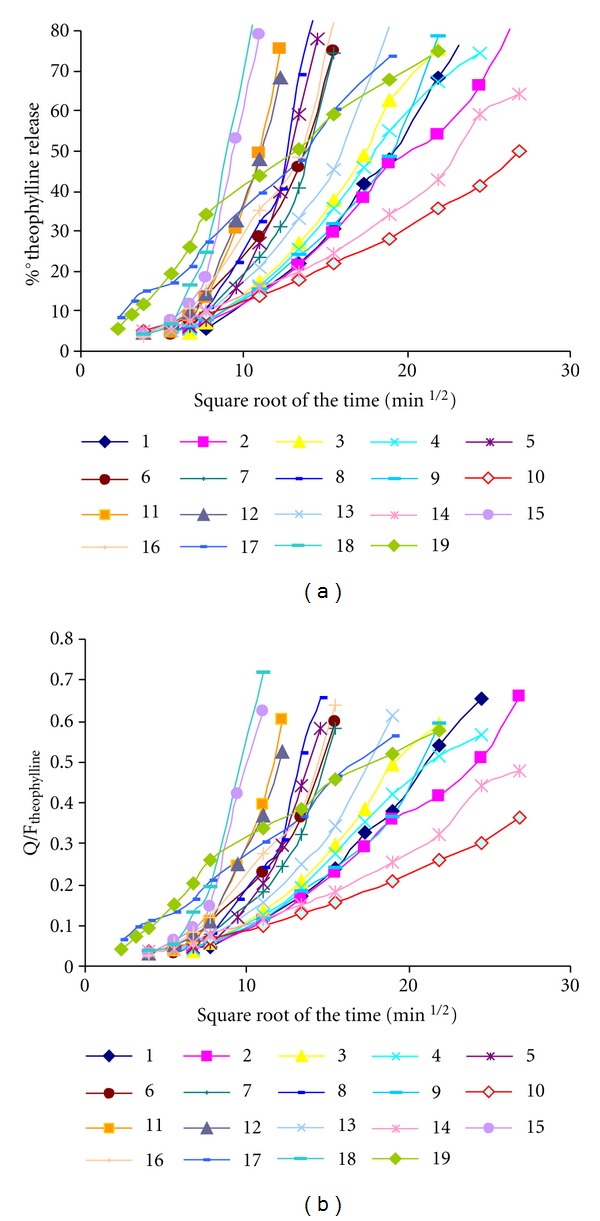
(a) Percentage of drug released versus square root of the time for the hydrophilic matrix systems. (b) Amount of drug released/volumetric fraction of the drug versus square root of the time for the hydrophilic matrix systems.

As it has been mentioned in the materials and methods section, the percentage of drug released and the amount of drug released normalized with the volumetric fraction of drug (Q/F_Theophylline_) has been plotted versus the square root of the time to show the different behavior of the matrices below and above the polymer percolation threshold. We can observe clearly different types of lines that reflect the differences between the different matrix systems studied (see Figure 6). 

Combined percolation thresholds have been studied and reported in ternary inert matrices by Caraballo et al. [15]. The degree in which these combined percolation thresholds can be appreciated depends on the influence that the discriminating property exerts on the property of the system is selected to calculate the threshold. As it has been mentioned in the introduction section, a discriminating property can be the solubility or the hydrophilicity of the substances. 

**Figure 7:**
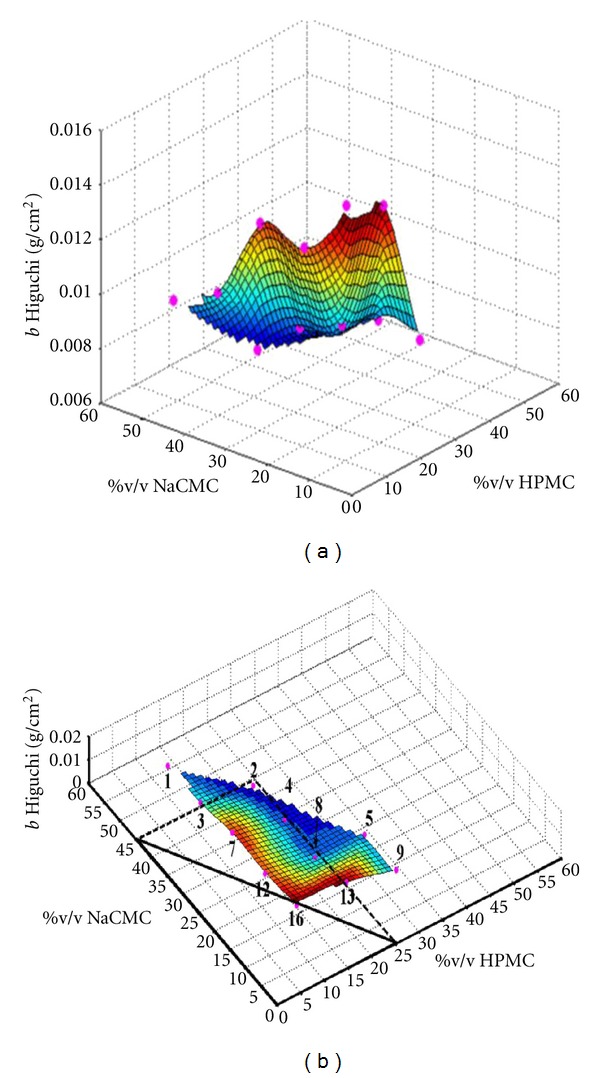
Response surface plot of the Higuchi's slope versus percentage of both excipient volumetric fraction for the ternary hydrophilic matrix systems 1, 2, 3, 4, 5, 7, 9, 12, 13, and 16.

In order to study the applicability of these concepts to the hydrophilic matrices studied in the present work, the obtained data are showed on a response surface plot using the program Matlab.  Figure 7 shows the evolution of one of the studied kinetic parameters, the Higuchi's slope, *b*, as a function of the volumetric fraction of both excipients (HPMC and NaCMC) at time zero. The batches plotted in Figure 7 are the ternary hydrophilic matrix systems containing 40 to 80% (w/w) theophylline. 

Two opposite patterns can be considered [16] in order to analyze this figure: 

Pattern 1: ∆ interchangeable excipients: this hypothesis supposes a full collaboration of both excipients in order to create the gel layer controlling the drug release from the hydrophilic matrices. In this case, the concentrations of the hydrophilic polymers HPMC and NaCMC will be fully additive, that is, the value of the Higuchi's slope, *b*, will be the same whenever the sum of the concentrations of both excipients will be the same, independent on the individual concentration of each one of the excipients. For example, we could expect the same release behaviour for a matrix containing 10% HPMC + 20% NaCMC than for another containing 25% HPMC + 5% NaCMC. 

In case that the obtained data would follow this pattern, the color lines in Figure 7 will show that the critical barrier is a straight line forming a triangle together with the *x* and *y*-axis.

Pattern 2: □ independent excipients: this hypothesis supposes that at least the critical concentration of one of the excipients (HPMC or NaCMC) has to be reached in order to obtain the gel layer controlling the drug release, independently on the concentration of the other excipient. In this case, the critical barrier would be formed by two straight lines with an angle of 90 degrees. They will form a square with the *x-* and *y*-axes.

In Figure 7, we can observe, for example, the behaviour of batches 7 and 9. The total polymer content is close to 42% (v/v) for both batches (see Table 3) and their Higuchi's slope values are 0.0135 and 0.009 g/cm^2^, respectively. Therefore, matrices having similar total polymer content show very different drug release behaviour. These data do not fulfil the hypothesis depicted in Pattern 1, indicating that the excipients are not interchangeable.

**Figure 8:**
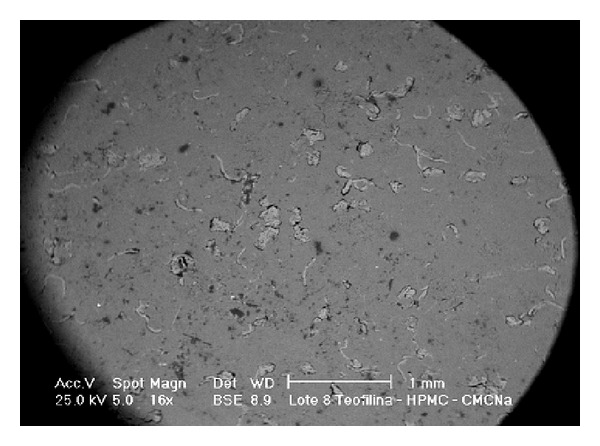
SEM micrograph obtained using a backscattering electrons (BSE) detector corresponding to the bottom side of a hydrophilic matrix from batch 8 (60% (w/w) theophylline: 20% (w/w) HPMC: 20% (w/w) NaCMC).

If we check the second hypothesis for batches 2 and 13, with similar HPMC concentrations (21.6% and 21.3%, resp.), the value of the Higuchi's slope is two times higher for batch 13 (see Table 3). Therefore, we can see that the release behaviour of the matrices with the same amount of one of the polymers is not independent on the concentration of the other polymer. This is indicative of collaboration between the two polymers in order to control the drug release. This collaboration is clearly appreciated in lot 4 and especially in lot 8 (the drug release is controlled for systems being below the percolation thresholds of the polymers). Nevertheless, the L-shaped drawing showed in Figure 7 indicates that the collaboration between the two polymers does not occur when the concentration of one of them is below 20% approximately (19.7% v/v).  Figure 8 shows an example of a hydrophilic matrix from lot 8. 

The need of a minimum concentration of a matrix forming polymer for establishing collaboration with other polymer has never been described before. Nevertheless, this could be an explanation for some studies showing unexpectedly low percolation thresholds for HPMC in matrix tablets containing important amounts of microcrystalline cellulose [22].

Furthermore, these findings are in agreement with the conclusions of the previous study dealing with matrices containing HPMC, NaCMC, and KCl as model drugs [16]. This previous paper reports a partial collaboration between the two polymers in order to establish the gel layer. The way in which this interaction affects the critical points was not described, probably due to the masking effect of the high solubility of KCl.

## 4. Conclusions

Applying the concepts of the percolation theory to binary theophylline and HPMC and theophylline and NaCMC hydrophilic matrices, the existence of critical points related to the excipient percolation threshold has been confirmed. The excipient percolation threshold for these binary hydrophilic matrices can be located between 21.5 and 31.3% (v/v) of HPMC and between 39 and 54% (v/v) of NaCMC, respectively.

It has been confirmed that HPMC and NaCMC do not show a combined percolation threshold for the whole range of concentrations. Nevertheless, it has been found for the first time that when the concentration of both excipients is above a minimum value (in our case this value is between 10 and 20% v/v approximately), there is an effective collaboration between the polymers. 

We are dealing with a new concept which is similar to the combined percolation threshold but includes the prerequisite of a minimum amount of the other polymer. In case that these findings are confirmed by further studies, they will help to design new solid dosage forms and to give an explanation to the behaviour of previously existing data.

## Figures and Tables

**Table 1:** Kinetic parameters for the zero-order, Higuchi, Korsmeyer, and Peppas-Sahlin models calculated for the binary and ternary hydrophilic matrix tablets.

				Zero order		Higuchi		Korsmeyer			Peppas and Sahlin		
Batch	%w/w Drug	%w/w HPMC	%w/w NaCMC	*k*_0_ (%min^−1^)	*r* ^ 2^	*b *(%min^−1/2^)	*r* ^ 2^	*k *(%min^−*n *^)	*n*	*r* ^ 2^	*k*_*d*_ (%min^−*m *^)	*k*_*r*_ (%min^−2*m *^)	*r* ^ 2^
10	60	40	0	0.078	0.982	2.127	0.978	0.553	0.703	0.998	0.927	0.107	0.998
14	70	30	0	0.108	0.987	2.406	0.938	0.354	0.819	0.997	0.524	0.159	0.997
17	80	20	0	0.270	0.994	4.693	0.960	1.696	0.679	0.994	2.311	0.254	0.996
19	90	10	0	0.350	0.979	5.585	0.995	2.019	0.676	0.999	2.627	0.307	0.999
6	60	0	40	0.429	0.927	7.934	0.880	0.002	2.025	0.999	−4.582	0.97	0.973
11	70	0	30	0.329	0.992	6.804	0.961	0.058	1.305	0.999	−2.414	0.648	0.998
15	80	0	20	0.425	0.999	8.047	0.981	0.119	1.231	0.999	−2.453	0.781	>0.999
18	90	0	10	0.465	0.956	5.268	0.911	0.015	1.816	0.999	−7.453	1.801	0.985
1	40	10	50	0.146	0.997	4.347	0.966	0.06	1.139	0.999	−1.741	0.453	0.999
2	40	20	40	0.113	0.988	3.492	0.986	0.206	0.906	0.997	−0.418	0.304	0.998
3	50	10	40	0.170	0.994	4.766	0.977	0.111	1.064	0.997	−1.235	0.38	0.998
4	50	20	30	0.132	0.977	4.123	0.987	0.273	0.887	0.995	−0.066	0.256	0.995
5	50	30	20	0.150	0.999	4.210	0.967	0.083	1.097	>0.999	−1.171	0.384	0.999
7	60	10	30	0.151	0.960	6.222	0.880	0.023	1.299	0.988	−1.54	0.323	0.984
8	60	20	20	0.187	0.994	4.447	0.955	0.12	1.076	0.998	−0.86	0.372	0.997
9	60	30	10	0.226	0.981	4.082	0.905	0.032	1.33	0.998	−2.501	0.556	0.993
12	70	10	20	0.286	0.997	6.070	0.977	0.156	1.108	0.999	−1.175	0.527	0.999
13	70	20	10	0.337	0.931	6.735	0.857	0.004	1.811	0.995	−3.88	0.775	0.975
16	80	10	10	0.340	0.978	7.023	0.942	0.085	1.247	0.994	−2.01	0.628	0.992

[*k*_0_ (%min^−1^): zero-order constant; b (%min^−1/2^): Higuchi's slope; *k* (%min^−*n *^): kinetics constant of the Korsmeyer model, *n*: diffusional exponent, *k*_*d*_: diffusional constant of Peppas and Sahlin model, *k*_*r*_: relaxation constant of Peppas and Sahlin model, *m*: exponent that depends on the geometric shape of the releasing device through its aspect ratio].

**Table 2:** Kinetic parameters for Davidsons and Peppas model calculated for the ternary hydrophilic matrix tablets.

Batch	*k*_s_ (%min^−*n *^)	*n*	*r* ^2^
1	8.91	0.643	>0.999
2	16.465	0.498	>0.999
3	24.804	0.498	>0.999
4	13.798	0.558	0.999
5	5.477	0.682	0.999
7	20.371	0.612	0.999
8	10.162	0.643	0.999
9	4.156	0.741	>0.999
12	9.288	0.643	0.999
13	26.886	0.588	>0.999
16	13.369	0.643	>0.999

*k*_s_ (%min^−*n *^): kinetic constant of water penetration; *n*: Exponent which depends on the water penetration mechanism.

**Table 3:** %v/v of the components theophylline, HPMC and NaCMC and Higuchi's slope values obtained for the ternary hydrophilic matrix systems.

Batch	%v/v theophylline	%v/v HPMC	%v/v NaCMC	Higuchi slope “ *b*” (g/cm^2^)
1	31.5	11.1	51.6	0.0095
2	30.8	21.6	40.3	0.0076
3	39.2	11.0	41.1	0.0104
4	38.3	21.5	30.1	0.0090
5	37.3	31.4	19.5	0.0092
7	47.3	11.0	30.9	0.0135
8	45.2	21.2	19.7	0.0097
9	45.2	31.7	9.9	0.0090
12	53.8	10.8	20.1	0.0132
13	53.0	21.3	9.9	0.0146
16	62.2	10.9	10.2	0.0153
